# Antagonist Concepts of Polypyrrole Actuators: Bending Hybrid Actuator and Mirrored Trilayer Linear Actuator

**DOI:** 10.3390/polym13060861

**Published:** 2021-03-11

**Authors:** Rudolf Kiefer, Ngoc Tuan Nguyen, Quoc Bao Le, Gholamreza Anbarjafari, Tarmo Tamm

**Affiliations:** 1 Conducting Polymers in Composites and Applications Research Group, Faculty of Applied Sciences, Ton Duc Thang University, Ho Chi Minh City 700000, Vietnam; lequocbao@tdtu.edu.vn; 2 Faculty of Applied Sciences, Ton Duc Thang University, Ho Chi Minh City 700000, Vietnam; nguyenngoctuan@tdtu.edu.vn; 3 iCV Research Lab, Institute of Technology, University of Tartu, 50411 Tartu, Estonia; shb@ut.ee; 4 Intelligent Materials and Systems Lab, Institute of Technology, University of Tartu, Nooruse 1, 50411 Tartu, Estonia; tarmo.tamm@ut.ee

**Keywords:** polypyrrole, polymerization conditions, linear actuators, bending hybrid actuator, mirror linear trilayer

## Abstract

Following the natural muscle antagonist actuation principle, different adaptations for “artificial muscles” are introduced in this work. Polypyrrole (PPy) films of different polymerization techniques (potentiostatic and galvanostatic) were analyzed and their established responses were combined in several ways, resulting in beneficial actuation modes. A consecutive “one-pot” electrosynthesis of two layers with the different deposition regimes resulted in an all-PPy bending hybrid actuator. While in most cases the mixed-ion activity of conductive polymers has been considered a problem or a drawback, here for the first time, the nearly equal expansions upon oxidation and reduction of carefully selected conditions further allowed to fabricate a “mirrored” trilayer laminate, which behaved as a linear actuator.

## 1. Introduction

The basic idea behind conductive polymer actuators has been the mimicking of natural muscles with the so called “artificial muscles” [1]. A lot of progress has been made in recent years in the field, aiming for potential applications in micro-actuators and robotics [2,3], by achieving high strains up to 34% [4] and stresses in range of 22 MPa [5]. There have been a number of different applications proposed [6] such as smart textiles [7], sensors [8], soft robotics [9], biomedical applications [10], and mechanically stimulated growth [11], and many more. The actuation principle of conductive polymers can be explained in a simplified manner by the formation of delocalized charges upon oxidation along the polymer chains, while ions (with solvent) move in to ensure charge neutrality. As a result, the conductive polymer as a free-standing film or a film deposited on flexible non-conductive substrate expands, creating linear or bending strain. The outcome depends on a variety of factors, including the polymerization procedure [12], temperature [13], electrolyte [14], solvent [15], osmotic pressure [16]. Having just a single ionic species controlling the actuation in one direction (oxidation or reduction) has been an important consideration as it would allow higher strains and consistent controllability, but in real world, mixed ion-activity is often observed instead, both in case of bending [17] or linear actuation [18].

As the motion of a natural arm is based on the alternating contraction of the antagonist muscles (biceps and triceps), several attempts have been made to replicate a similar principle using artificial muscles, where one side contracts while the other extends in length [19,20].

Here, our aim was to attempt to apply the fact that deposition conditions radically influence the actuation properties of PPy films for fabricating a bending actuator by combining a layer of potentiostatically deposited film (PPy(pot)) with a galvanostatically deposited one (PPy(galv)) into a freestanding bending hybrid PPy actuator (BHA) synthesized from one pot. In order to tune the individual layers, the films were first studied separately. A similar concept has been employed by combining different PPy films in aqueous electrolyte [21] as well as for PEDOT films polymerized at different polymerization potentials [22]. Recently, PEDOT IPN hollow fibers operating in air [23] and other cases have been considered, where one electrode with high deformation is coupled with another with minimal deformation [24].

Another completely novel (antagonist) approach introduced here is to take advantage of a feature of conductive polymers that is usually considered undesirable–the mixed ion activity. A trilayer with an inert separator in between two PPy layers can function as a linear actuator, as long as equal expansion/contraction upon both oxidation and reduction, a mirrored (PPy(mirr)) design can be achieved.

Cyclic voltammetry (scan rate 5 mV s^−1^) and square potential step driven actuation measurements in frequency range of 4.17 mHz to 0.1 Hz were performed. Characterization of the PPy films was made by scanning electron microscopy (SEM), energy dispersive X-ray (EDX) spectroscopy was carried out to study the mobile ionic species.

## 2. Material and Methods

### 2.1. Materials

Tetrabutylammonium trifluoromethanesulfonate (TBACF_3_SO_3_), ethanol (technical standard) and propylene carbonate (PC, 98%) were purchased from Sigma-Aldrich (Taufkirchen, Germany) and used as received. Pyrrole applied as the monomer in electropolymerization (Py, 98%, Sigma-Aldrich) was distilled and stored at low temperature under nitrogen in the dark. Polydimethylsiloxane (SYLGARD^®^ 184, 6500 cSt) two component system was purchased from Sigma-Aldrich and applied as the separator layer in the mirrored linear actuator design.

### 2.2. Electropolymerization

**Figure 1:**
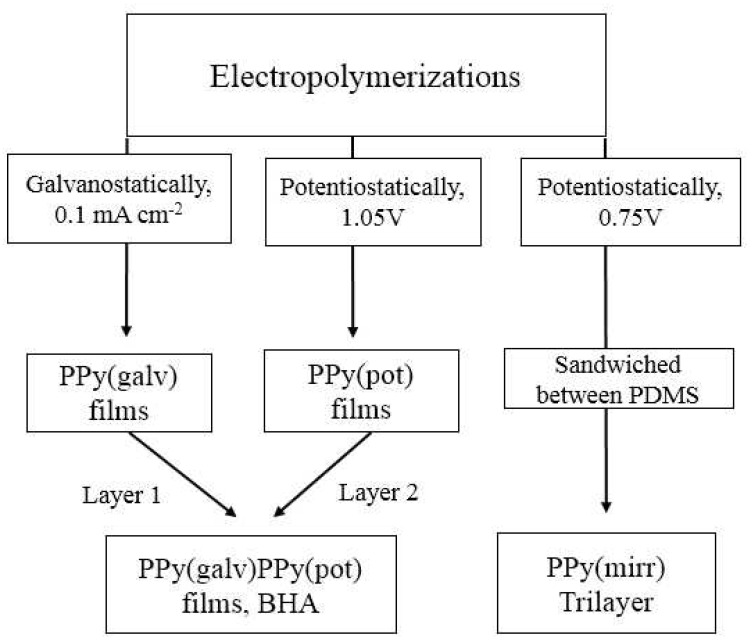
Flowchart of the fabrication of the various types of actuator materials, including pyrrole electropolymerization regimes.

The electropoymerizations were carried out in a three-electrode cell using a stainless steel sheet (12 cm^2^) as the working electrode, a Ag/AgCl wire as the reference electrode (+0.15 V against Ag/AgCl (3 M KCl)), and a platinum mesh counter electrode at −20 °C. Figure 1 represents a flowchart of the principle electropolymerizations.

The monomer solution consisted of 0.1 M Py, 0.1 M TBACF_3_SO_3_ dissolved in propylene carbonate. Galvanostatic electropolymerization was performed at 0.1 mA cm^−2^ (5.5 h, charge density 0.675 C cm^−2^) leading to 15 ± 1.2 μm PPy(galv) films (Figure 1). The potentiostatic polymerization (1.05 V vs. Ag/AgCl wire, about 1.2 V against SHE) was carried out for 10.3 h to consume the same charge density of 0.675 C cm^−2^ leading to PPy(pot) films in thickness of 15.2 ± 1.5 μm (Figure 1). The bending hybrid actuators (BHA) (Figure 1) were obtained by the galvanostatic electropolymerization of PPy on stainless steel (0.1 mA cm^−2^, 5.7 h, −20 °C, 15.6 ± 1.3 μm) named as layer 1 followed by potentiostatic polymerization (1.05 V, 11 h, 15.9 ± 1.6 μm) on top of the PPy(galv) (layer 2) resulting in films with thickness of 31.5 ± 2.2 μm (Figure 1). For mirrored trilayers, free standing PPy films were obtained in potentiostatic polymerization at 0.75 V (0.9 V against Ag/AgCl (3 M KCl)) in the same monomer solution at −20 °C for 14 h yielding 14.5 ± 1.1 μm thick films with the consumed charge density of 0.675 C cm^−2^. After polymerization, all films were washed with ethanol to remove PC and monomer, dried in oven at 4 mbar for 12 h at 40 °C.

For trilayers, the two-component PDMS mix was prepared and coated on glass slides in thickness of 150 μm, one PPy free standing film (thickness 15 μm) was attached on top before the silicone dried up. After removing PDMS-PPy from the glass slide, the other side was coated with pre-polymer mix of Sylgard in thickness of 50 μm and before drying, the other PPy film (15 μm) was attached to it, forming a trilayer (PPy-PDMS-PPy, PPy(mirr), ~230 μm) (Figure 1).

### 2.3. Actuation Measurements

**Figure 2:**
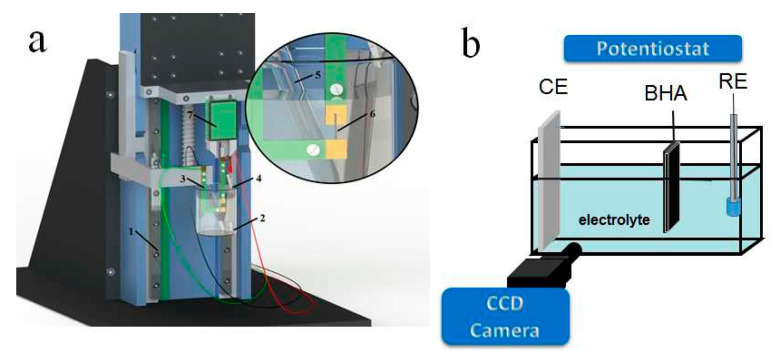
(**a**) Electro-chemo-mechanical deformation (ECMD) device with linear actuation stage (1), beaker with electrolyte (2), electrodes of the potentiostat (3–5), measured samples such as freestanding PPy films of PPy(galv), PPy(pot) and PPy(mirr) (6) and force sensor (7) [25]. The bending hybrid actuator (BHA) was studied in a three electrode cell shown in (**b**) with counter electrode CE, reference electrode RE, the displacement was recorded with a CCD.

Figure 2a,b show the measurement set ups applied for different designs of actuators.

PPy(galv), PPy(pot) and PPy(mirr) samples were cut in strips of 1 cm length, 2 mm width and fixed between contacts of the force sensor and the lower clamp (4 mm free length, constant force of 9.8 mN) in a three-electrode set up shown in Figure 2a.

For the free-standing films, the working electrodes were the PPy films and the counter electrode a stainless steel sheet, while for PPy(mirr), one side was connected as the working and the other as the counter electrode. In all cases a Ag/AgCl (3 M KCl) reference electrode was used in the linear measurement set up. From the ECMD set up (Figure 2a), the force changes were translated in movement by an in-house computer program, as described in detail previously [25].

Length change measurements (strain ε = ΔL/L with ΔL = L − L_1_ (L the initial free length of film, and L_1_ the actuated length) were performed under cyclic voltammetry (±1 V, 5 mV s^−1^) and square wave potential steps (±1 V, frequency from 0.00417 Hz to 0.1 Hz) controlled by a potentiostat (Biologic PG581, Seyssinet-Pariset, France), in 0.1 M TBACF_3_SO_3_ PC electrolyte solution. Before the measurements, the samples were stored overnight in the electrolyte in stretched position of 1% strain for PPy(galv) and PPy(pot) and at 0.5% strain for PPy(mirr).

The BHA in length of 2.5 cm (of which 2 cm was in the electrolyte), width of 0.4 cm and thickness in range of 31.5 ± 1.5 μm consisting of the PPy(galv) and PPy(pot) layers was connected as the working electrode, with a platinum mesh counter electrode and a Ag/AgCl (3 M KCl) reference electrode in 0.1 M TBACF_3_SO_3_ PC electrolyte solution (Figure 2b). The BHA films were fixed with a metal clamp hanging in electrolyte solution for 12 h before the measurements. The bending displacement was recorded with a CCD camera (Cyber-shot DSC-F717, Sony, Tokyo, Japan) as movies (Figure 2b), which were converted to images whereas the bending was determined over angular motion as described previously [26]. Square wave potential steps were performed with a frequency scan between 4.17 mHz to 0.1 Hz in the potential range 1 V to 0 V.

To determine the diffusion coefficients *D*, Equations (1) and (2) were applied on the current density time curves of square wave potential step measurements [27]:

(1) $$ln\left[1-\frac{Q}{Q_{t}}\right]=-bt$$

(2) $$D=\frac{bh^{2}}{2}$$

The approach originated from the Cottrell model, where the determination of diffusion coefficients was made under the assumption that the polymer has a porous open structure and no structural or conformation change occur during electrochemical redox process, which does not hold in case of PPy. The incorporation of the structural changes during redox processes from the electrochemically stimulated conformational relaxation (ESCR) model [28] is expressed in Equations (1) and (2). The model development from the chronoamperometric response has been described in details by Suarez et al. [29]. The term *Q*/*Q_t_* on the left side of the Equation (1) was obtained by the integration of current density time curves, where Q is the charge density at each timepoint, and is *Q_t_* the total charge density. The term ln [1 − *Q*/*Q_t_*] was plotted against time *t*, and the slope *b*. The diffusion coefficients were calculated from Equation (2) by including the thickness h of the samples. The hypothesis of the Equations (1) and (2) is that upon oxidation the polymer expands and upon reduction it shrinks, with kinetic conformational changes leading to compaction. Diffusion coefficients for expansion from compact form (reduced state) upon oxidation can be calculated from Equations (1) and (2) under the conditions of steady state (charging/discharging in balance). The Equations (1) and (2) relate to the diffusion coefficients of the polymer-electrolyte interface as well as of ions inside the polymer films.

### 2.4. Characterization

Surface and cross-section scanning electron microscopy (SEM, Helios NanoLab 600, FEI, Hillsboro, OR, USA) images were recorded (at 5 kV) after polymerization from washed and dried films The ion content in oxidized (5 min, +1 V) and reduced state (5 min, −1 V) were determined in the cross-sections of PPy(pot) and PPy(galv) samples using an energy dispersive X-ray spectroscopy system (EDX with X-Max 50 mm^2^ detector, Oxford Instruments, High Wycombe, UK). The surface conductivity of the samples was measured over four-point-probe conductivity meter (Jandle 4-Point Probe Head, Model RM2, Leighton Buzzard, UK).

## 3. Results and Discussion

**Figure 3:**
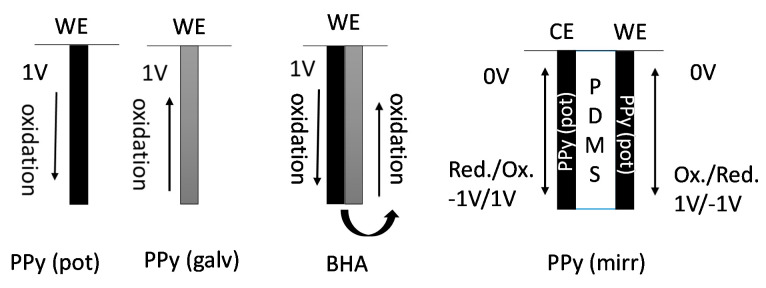
PPy(pot) and PPy(galv) in different arrangements: as freestanding films, BHA (bilayer) and linear trilayer actuator (PPy(mirr)). WE refers to the working electrode, CE to counter electrode. The arrows symbolize expansion or contraction under given conditions.

In order to understand the effects of direction of actuation and the dominant mobile species, the PPy(pot) and PPy(galv) freestanding films were investigated in their linear actuation properties. The understanding gained allowed to combine these two films to form pure-PPy bending actuators as well as linear trilayer actuators. For the first time, the usually undesirable mixed ion actuation was employed to make use of the virtually equal strain upon oxidation/reduction in a linear trilayer actuator design. The different actuation models consisting of these two films are shown in Figure 3.

From each model (Figure 3) in different arrangements, at least three different samples were fabricated and tested, the actuation results are presented as mean values with standard deviations. It need be noticed that the PPy(pot) and PPy(galv) represents freestanding films, while BHA is essentially a bilayer of two PPy layers consecutively electropolymerized with the two techniques. PPy(mirr) is a trilayer with identical potentiostatically deposited PPy layers on either side of a PDMS separator. During oxidation/reduction the trilayer (PPy(mirr)) expands and contract in cycles at 0 V shown in Figure 3.

### 3.1. Characterization

**Figure 4:**
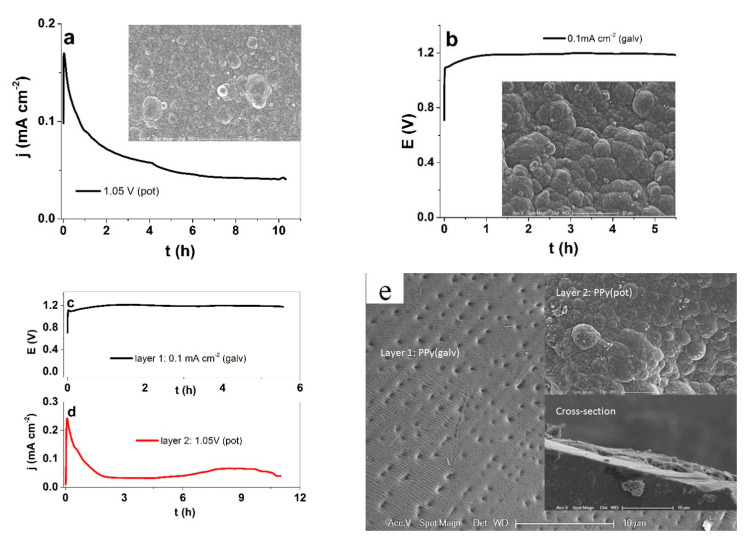
Electropolymerization curves and SEM surface images (scale bar 10 µm) in (**a**) potentiostatic at 1.05 V (vs Ag/AgCl wire) and (**b**) galvanostatic at 0.1 mA cm^−2^. The electropolymerization of the PPy for BHA: (**c**) layer 1, galvanostatic (0.1 mA cm^−1^) on stainless steel (black curve); and in (**d**) layer 2, potentiostatic (1.05 V) (red curve). The SEM surface images (scale bar 10 µm) of BHA (**e**) PPy(galv) layer 1 with insets layer 2 (PPy(pot)) and cross-section.

The potentiostatic (1.05 V) and galvanostatic electropolymerization curves of PPy are shown in Figure 4a,b, respectively, with SEM surface images as insets. Figure 4c,d show the polymerization curves of the galvanostatic layer 1 and the potentiostatic (1.05 V) layer 2 of BHA, respectively. The SEM image of the surfaces of the BHA opposite sides and the cross section (inset) are shown in Figure 4e.

**Figure 5:**
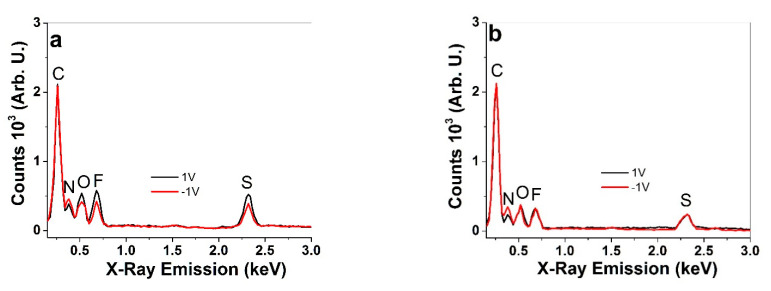
EDX spectra of the cross-sections of PPy films in oxidized state (1 V, 5 min polarization, black line) and at reduced (−1 V, 5 min polarization, red line) (**a**) PPy(pot) and (**b**) PPy(galv).

To be able to compare differently deposited materials, some parameters need to be kept constant. To make sure that a similar amount of PPy was deposited with the two methods, the consumed charge density of 0.675 C cm^−2^ was kept constant. The current density decreased during the potentiostatic polymerization from nearly 0.12 mA cm^−2^ to 0.044 mA cm^−2^ by the end of the polymerization (11 h), as with increasing film thickness (and resistance), the relative driving force of the potential dropped. The PPy(pot) film conductivity was in range of 7.8 ± 0.7 S cm^−1^. In case of the galvanostatic electropolymerization (constant current density of 8.3 μA cm^−2^), the potential remained rather constant in range of 1.18 V, the conductivity of the obtained PPy(galv) film was 8.5 ± 0.6 S cm^−1^. On the SEM surface images, both types of PPy films appear relatively similar, with the typical cauliflower morphology [30]. The latter is logical, as both current and potential (reaction rate and driving force) remained virtually constant throughout the synthesis. To obtain the BHA, the first layer was deposited galvanostatically on stainless steel, with a similar voltage evolution reaching 1.19 V by the end of polymerization. Layer 2 was then polymerized potentiostatically (1.05 V) on top of layer 1. The current density again dropped from the peak of 0.2 mA cm^−2^ to nearly 0.042 mA cm^−2^ by the end. Here, the SEM surface images look more distinct, as the galvanostatic one was peeled from the stainless steel sheet and looks much smoother while the potentiostatic one closely resembles the one of the free-standing PPy(pot) film. However, the cross section shows no distinctive separation or difference between the two layers. The conductivities were also relatively similar, for the galvanostatic and potentiostatic layers, respectively. It is expected that the different deposition conditions also reflect on the polymer structure, and hence the ion transport within. To determine the dominant mobile ionic species in PPy(pot) and PPy(galv) free standing films, EDX spectroscopy was performed in the oxidized and reduced states (Figure 5).

In all spectra, the carbon peak (C) at 0.26 keV, the nitrogen (N) peak at 0.38 keV, the oxygen peak (O) at 0.52 keV, the fluorine peak (F) at 0.68 keV and the sulfur (S) peak at 2.32 keV can be seen. Upon oxidation, the sulfur, fluorine and oxygen peaks of potentiostatically polymerized PPy (Figure 5a) increase, which refers to the incorporation of solvated CF_3_SO_3_^−^ anions during oxidation. In the reduced state, a certain amount of CF_3_SO_3_^−^ anions still remain in the film. It has been shown previously [31,32] that some CF_3_SO_3_^−^ anions become immobile in a PPy network due to their non-spherical form, forcing (solvated) cation ingress upon reduction to maintain electroneutrality, as indicated here with a slight increase of the nitrogen peak. Nitrogen peak intensity is the only one changing in the spectra of the galvanostatically polymerized PPy(galv) (Figure 5b), indicating virtually pure cation activity, which has been observed before for similar films [33].

### 3.2. Comparison of PPy(galv) and PPy(pot) Actuation

**Figure 6:**
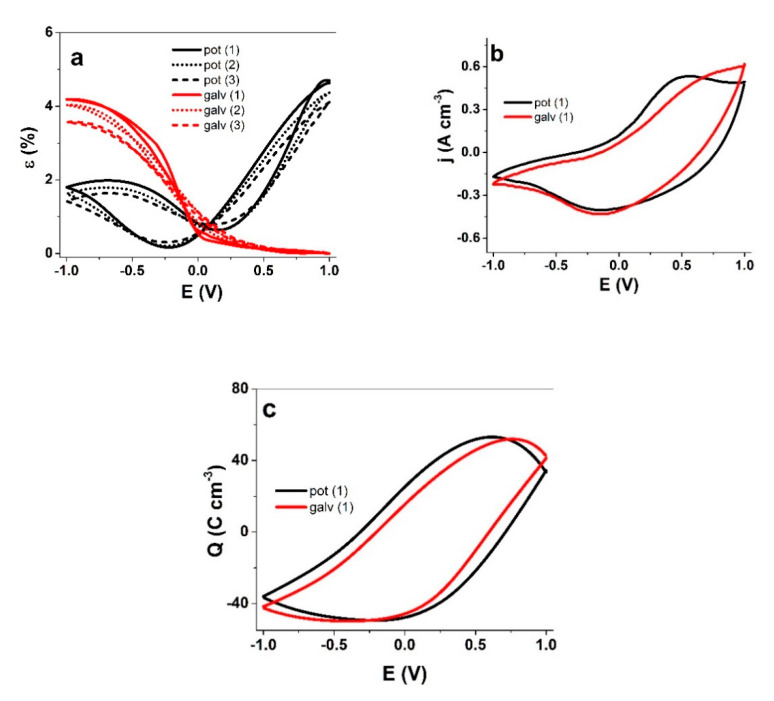
Cyclic voltammetry (±1 V, 5 mV s^−1^) driven linear actuation of PPy(pot) (black) and PPy(galv) (red) in a three-electrode cell with TBACF_3_SO_3_-PC solution The working electrode was the samples, counter electrode a platinum sheet and reference electrode a Ag/AgCl (3 M KCl). (**a**) strain ε of cycles (1) solid, (2) dotted, (3) dashed line; (**b**) current density j; (**c**) charge density Q against the potential E. The CV and charge density curves correspond to the strain curve (1).

The actuation behavior of the PPy(pot) and PPy(galv) films in TBACF_3_SO_3_ PC solution driven by cyclic voltammetry (scan rate 5 mV s^−1^) was studied (Figure 6). To make sure that charging/discharging was in balance [34] the potential range of ±1 V was applied.

The strain response of PPy(pot) and PPy(galv) reflected their mobile ionic species, as established from the EDX spectra. For PPy(pot), a mixed response was observed, with 4.7% strain upon oxidation and 1.8% upon reduction. PPy(galv) was almost purely cation-active, with strain upon reduction in range of 4.2%. For the latter case, the chosen potential range likely plays a role as well [33]. With such differences in the strain response, it is perhaps surprising that the current density curves look so similar at first glance, especially on the reduction side. However, upon closer observation it is possible to distinguish an oxidation peak at 0.5 V in addition to the reduction peak at −0.15 V for PPy(pot), while PPy(galv) had just the reduction peak.

**Figure 7:**
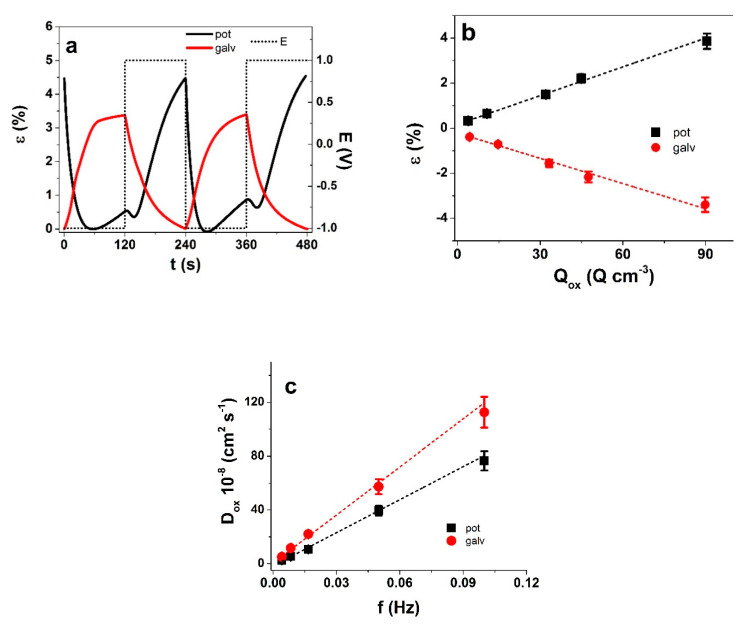
Response to square wave potential steps in TBACF_3_SO_3_ PC of PPy(pot) (black) and PPy(galv) (red) (**a**) strain vs. time (2 subsequent cycles 3rd–4th) at ±1 V (dotted) and 4.17 mHz. (**b**) strain vs. charge density Q_ox_ on oxidation; (**c**) diffusion coefficients upon oxidation D_ox_ obtained from Equations (1) and (2) vs. frequency f. The dashed lines in (**b**,**c**) represent linear fits and are shown here only for orientation. Negative values of strain in b represent opposite actuation direction.

The similarity in the charge density but not in strain reveal clear differences in the electro-chemo-mechanical coupling—the flow of current in PPy(galv) coupled with ion flux generates a lot more mechanical stress in the polymer matrix, resulting in higher displacement (Figure 6a,b). The charge density curves revealed for both PPy films a close loop which shows that charging/discharging was in balance [34] and no irreversible processes took place (Figure 6c). The charge densities for PPy(pot) and PPy(galv) were found in similar range—101.6 ± 8 C cm^−3^ and 100.8 ± 7 C cm^−3^, respectively. While PPy is a typical faradaic actuator material where charge density should determine the actuation extent, it only forms the basis, and the understanding of the coupling and mobile species is needed for a more complete picture. In comparison to the gradual potential sweep of the cyclic voltammetry technique, the abrupt nature of square wave potential step signals creates responses further away from equilibrium. The response of PPy(pot) and PPy(galv) films to such measurements (at 4.17 mHz) are shown in Figure 7. The current density time curves (Figure S1a) correspond to the strain response in Figure 7a.

As for cyclic voltammetry, the two PPy materials showed contrasting actuation response. PPy(pot) had main expansion on oxidation in range of 4.5% and a small expansion on reduction, around 0.73%, from cycle to cycle, the strain increased slightly (Figure 7a). The current density (Figure S1a) showed a smaller second maximum, which indicates the start of another process, most likely related to the mixed ion transport. In case of PPy(galv), the expansion was again found on reduction, in range of 3.3% strain and there was no second peak in the current density curves either. The strain of both types of films followed the well-established inverse frequency dependence (Figure S1b). The charge density was obtained from each chronopotentiograms by integration at each frequency. As the square wave potential steps approach allows little time for relaxation, there was nearly linear dependence of strain (negative values for PPy(galv)) on charge density (Figure 7b), as expected for a faradaic actuator.

For both types of PPy films, the diffusion coefficients on oxidation (Figure 7c) increased with increasing frequency, which is the result of the timeframe for the process. Longer oxidation times allow deeper/more complete shrinking/compaction/relaxation/swelling [35]. The diffusion coefficients on oxidation of PPy(galv) were 1.5 to 2 times higher in comparison to PPy(pot), which is explained by the different ionic species participating in the charge compensation. In PPy(pot) upon oxidation the cations are expelled while anions also move in, while in PPy(galv) the charge neutrality is obtained primarily by cation expulsion. As indicated by the results with the free-standing PPy(pot) and PPy(galv), the different deposition conditions lead to similar charge density upon oxidation with opposite strain, and significantly higher diffusion coefficients for PPy(galv). Table 1 sums up the main characteristics of the PPy(pot) and PPy(galv) freestanding films.

Longer-term measurements (100 cycles, not shown here) at 0.1 Hz revealed that both types of PPy films had nearly equal stability as well, therefore, the materials can be combined into various beneficial hybrid actuator designs, as demonstrated below.

### 3.3. Actuation of BHA

**Figure 8:**
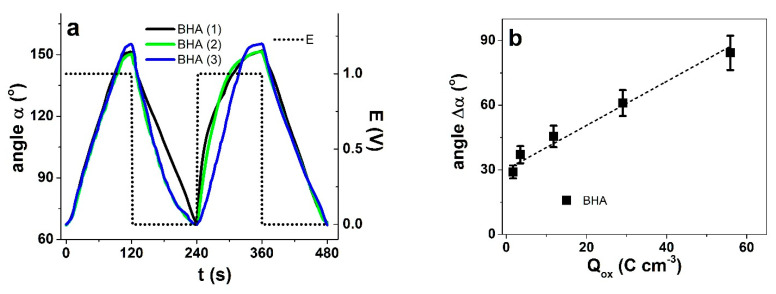
(**a**) Bending displacement of BHA (repeatability shown by three different samples: black, green and blue) in TBACF_3_SO_3_ PC as angle α at 4.17 mHz, with potential E (dotted) at 0 V to 1 V showing 2 subsequent cycles and in (**b**) Displacement of BHA (■) as angle change Δα against charge density upon oxidation Q_ox_ in potential range 0 V to 1 V. The dashed line in (**b**) represents the linear fit, shown here for orientation only.

**Figure 9:**
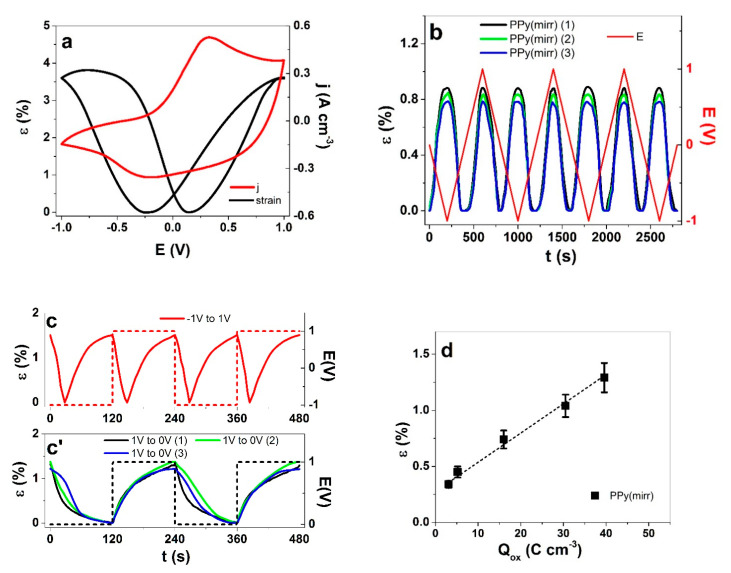
Linear actuation response to cyclic voltammetry (5 mV s^−1^) in 0.1 M TBACF_3_SO_3_ PC as strain (black) and current density (red) of (**a**) PPy(pot, 0.75 V) and (**b**) PPy(mirr) (repeatability shown by three samples: black, green and blue) against time t with applied potential E (dashed, red). The PPy(mirr) strain response to square wave potential steps (cycles 3rd–4th) at 4.17 mHz against time t (**c**) potential range from −1 V to 1 V (red) and (**c’**) potential 0 V to 1 V (red line) with strain response of three samples (black, green and blue). Strain ε (■) against charge density Q_ox_ in potential range 0 V to 1 V is shown in (**d**). The dashed line in (**d**) represents the linear fit and is shown for orientation only.

Since the PPy(galv) and PPy(pot) showed opposite actuation direction, it should be possible to fabricate a purely PPy-based bending actuator from the same synthesis solution. The BHA bilayer actuators were fabricated by depositing PPy in two consecutive steps applying the two methods showing electronic conductivity of the PPy(galv) side of 7.7 ± 0.7 S cm^−1^ and at the PPy(pot) side in similar range of 7.5 ± 0.5 S cm^−1^. In order to take maximum advantage of the bilayer design (length 2 cm, width 0.4 cm and thickness 31.5 ± 1.5 μm), a potential range must be chosen where one layer has significant displacement, while the other has no expansion or contracts at the same potential. The images of bending with angle determination at 0 V to 1 V at 4.17 mHz are shown in Figure S2a,b, the corresponding bending angles against time are presented in Figure 8a. The angle differences Δα against frequency in potential range 1 V to 0 V are shown in Figure S3, and those against the consumed charge density in Figure 9b. At least three independent samples from each polymerized BHA were studied, the angles in Figure S3 and Figure 5b are presented as mean values with standard deviations.

The actuation response of BHA in repeated sample 1–3 clearly depended on the chosen voltage range. At 0 V to 1 V range, mainly triflate anions were exchanged leading to an expansion of the PPy(pot) layer and the BHA bends to right, towards PPy(galv) (Figure S2a,b), thus, the BHA works like a bilayer. If more negative potentials were applied, irreversible damage to the BHA was observed, destroying the bending response (images available in Figure S3). The displacement in 0 V to 1 V range was 84 degrees. Upon reduction to −1 V, a displacement of 126 degrees was reached, however, no further displacement response was achieved with following charging/discharging cycles, the BHA stayed immobile. A similar observation has been reported for PEDOT bilayers [22]. Therefore, to obtain reversible bending from cycle to cycle, the 0 V to 1 V potential range was chosen.

The dependence of bending angle difference on frequency is shown in Figure S4 and on the consumed charge density in Figure 8b. As expected, the actuation depended virtually linearly on charge density, reaching 84 ± 2 degrees at the highest charge density of 56 ± 5 C cm^−3^ (corresponding to the lowest frequency 4.17 Hz) but still maintaining significant actuation of 30 ± 2 degrees at the highest frequency (1 Hz) which corresponded to just 1.7 ± 0.1 C cm^−3^ exchanged. Therefore, both charge efficiency and response speed of BHA were significant. The maximum bending angle naturally depends on several factors, including sample thickness, driving voltage and regime etc., rendering direct comparisons to other works difficult. While the respectable response even at higher frequencies suggests this type of bending actuators could have potential application in smart devices, the durability and creep issues need to be established first. The latter was noticeable at the highest frequencies considered. Long term measurements (not shown here) of 1000 cycles at 1 Hz showed from cycle 5 an angle of 30 ± 2 degree and decreased to 27 ± 2 degree assuring relatively good stability.

### 3.4. Mirrored Trilayer

For practical designs, self-contained linear actuators would be far more desirable than bending ones. In order to transfer a typical bending trilayer to a linear actuator, the active sides should have mirrored-response to voltage—one that expands/elongates upon oxidation and the other that expands/elongates upon reduction. This should all happen in the same electrolyte and to a similar extent under similar (opposite sign) polarization. To our knowledge, the present work is the first attempt to make use of the mixed-ion involvement in conductive polymers to achieve such a result. As no membrane is needed for electrolyte storage if the actuator is operated immersed in solution, the two active layers—two PPy(pot) free-standing films polymerized at 0.75 V (polymerization curve and SEM images shown in Figure S5a,b) were glued together with a two-component silicone, forming a trilayer PPy(pot)-PDMS-PPy(pot). Figure 9 shows the response of the free-standing films as well as the trilayer to both CV and square wave potential steps.

The potentiostatic electropolymerization at 0.75 V resulted in a film that showed nearly equal peak strain in both oxidation and reduction of 3.6% and 3.63%, respectively (Figure 9a). The lower polymerization potential resulted in a slightly higher conductivity of the films, in range of 13.4 ± 1.1 S cm^−1^. The CV shape showed an oxidation peak at 0.33 V and a reduction peak at −0.25 V. Comparing these ones to the PPy(pot, 1.05 V) films (Figure 6a), the lower formation potential of PPy(pot, 0.75 V) also resulted in a more compactly packed film (Figure S5b, inset), clearly limiting the mobility of the triflate anions, leading to a significant increase of cation participation in maintaining the charge neutrality.

In trilayer configuration (Figure 9b), the freedom of motion is limited by the (relatively thick) separator layer, resulting in lower strain of just 0.84 ± 0.05% upon polarization, but importantly a symmetrical linear strain upon oxidation and reduction is obtained together with high repeatability, while the contraction takes place at 0 V. The result proves that by achieving a balanced mixed ion involvement, linear actuation of trilayers with the exact same materials for electrodes can be obtained. The more abrupt driving by square potential steps in range of ±1 V at 4.17 m Hz (Figure 9c) pushed the trilayer to an increased strain of 1.5%, while maintaining the symmetric behavior. While a full range of potentials expectedly resulted in undesired double actuation, the potential range of 0 V to 1 V (Figure 9c’) was chosen for further analysis with maximum strain of 1.3 ± 0.08%. The strain against frequency is shown in Figure S6 and strain against charge density in Figure 9d. The nearly linear dependence of strain on charge density again attests a faradaic process [35]. As a summary, two PPy films having virtually equal strain response upon oxidation/reduction by taking advantage of the mixed ion activity can be combined with an inert separator to form a mirrored linear actuator PPy(mirr)). The mirror-like actuation of PPy(mirr) trilayers might find applications in micro-pumps or soft robotic devices. By replacing the PDMS layer with a porous membrane and choosing appropriate electrolytes, such trilayer linear actuators could also be designed to be self-contained and to operate in air/vacuum.

Table 2 shows the comparison of the properties of PPy films in the two different arrangements of BHA and PPy(mirr).

Actuators of both designs showed good repeatability. Further research on their adaptability in different application is envisaged.

## 4. Conclusions

While it is a well-known fact that the synthesis conditions affect the properties of conductive polymers, surprisingly few attempts have been made to tune the conditions like the electropolymerization regime for making hybrid actuators. Moreover, mixed ion involvement has been usually considered an adverse effect that has to be minimized or best avoided. Here we have demonstrated that as the potentiostatically and galvanostatically polymerized polypyrrole films and have rather different actuation responses, the former showing mixed ion activity while the latter is primarily cation-active and various beneficial antagonist designs can be created. For instance, it is possible to create a bending actuator by a two-step PPy deposition from just one polymerization solution, with no additional fabrication or addition of passive layers. In this example, the main work was done by the potentiostatic PPy layer, which in the voltage range of 0 V to 1 V primarily exchanges anions, while the galvanostatic PPy layer shows limited cation activity, but here behaves as a virtually passive layer turning the actuation into bending. Moreover, by exploiting the response sensitivity towards potentiostatic polymerization potential, it is possible to synthesize films with nearly equal cation and anion activity. By combining such films into a trilayer, a mirrored actuator is obtained with linear actuation response, as both layers are capable of expansion/elongation upon opposite polarizations. For the first time, equal anion and cation involvement has been put into good use. Both the possibility for simple fabrication of bending actuators or creating linear trilayer actuators can open up new applications in soft robotics and smart fabrics.

## Figures and Tables

**Table 1:** Strain (negative strain refers to expansion on reduction), charge density on oxidation Q_ox_, and diffusion coefficient on oxidation D_ox_ at 16.7 mHz with sample dimensions and conductivities of PPy(galv) and PPy(pot) free-standing films (mean values).

Free-Standing Films	Strain ε (%)	Q_ox_, (C cm^−3^)	D_ox_ 10^−8^ (cm^−2^ s^−1^)	Conductivity (S cm^−1^)	l (mm) × w (mm) × d (μm)
PPy(galv)	−1.56 ± 0.16	33.2 ± 0.3	22.0 ± 0.20	8.5 ± 0.6	10 × 2 × 15.0 ± 1.2
PPy(pot)	1.49 ± 0.14	32.0 ± 0.2	10.6 ± 0.11	7.8 ± 0.7	10 × 2 × 15.2 ± 1.5

**Table 2:** Comparison of the main characteristics of BHA and PPy(mirr): actuation behavior, charge density upon oxidation during square wave potential steps from 0 V to1 V at 16.7 mHz, conductivity and dimensions (length l, width w and thickness d).

Design	Actuation	Charge Density (C cm^−3^)	Conductivity (S cm^−1^)	Dimension (l × w × d)
BHA PPy(galv)-PPy(pot, 1.05 V) bilayer	45.5 ± 4 degree bending	11.8 ± 1.1	~7.6 ± 0.7	20 mm × 4 mm ×31.5 ± 2.2 μm
PPy(mirr) PPy(pot 0.75 V)-PDMS-PPy(pot, 0.75 V) trilayer	0.74 ± 0.08% linear	16 ± 1.5	13.4 ± 1.1	10 mm × 2 mm × 230 ± 2 μm

## Data Availability

The data presented in this study are available on request from the corresponding author.

## Supplementary Materials

The following are available online at https://www.mdpi.com/2073-4360/13/6/861/s1, Figure S1: a: The charge density j against time t of PPy(pot) (black line) and PPy(galv) (red line) films at frequency 0.00417 Hz showing 2 subsequent cycles in TBACF3SO3 PC electrolyte against potential E (dotted) of ±1 V. The strain (positive refers to anion driven and netaive to cation-driven) against frequencies f (0.00417 Hz–0.1Hz) of PPy(pot) (■) and PPy(galv) (●) are presented in (b), Figure S2: Images of the bending hybrid actuator (BHA) consist of PPy(pot)PPy(galv) polymerized films operated in TBACF3SO3 PC electrolyte in a three electrode cell with platinum counter electrode (CE) and a Ag/AgCl (3M KCl) reference electrode (RE) of BHA (left side the PPy(pot) and right side the PPy(galv))in potential range 0 V to 1 V at 0 V in (a) and at +1 V in (b), Figure S3: Images of BHA in square wave step measurements at applied frequency of 4.17 mHz showing images in a: at 0 V, b: at 1 V and c: at −1 V. The bending displacement in angle against the time t are shown in (d) with the applied potential E, Figure S4: The displacement difference in angle Δα against frequencies f (4.17 mHz–1 Hz) in potential range 1 V to 0 V are shown of the BHA samples in 0.1M TBACF3SO3 PC electrolyte, Figure S5: a: Current density time curve of PPy polymerized potentiostatically at 0.75 (at −20 °C, in 0.1 M pyrrole and 0.1 M TBACF3SO3 PC) against Ag/AgCl wire (equal 0.9 V against Ag/AgCl (3M KCl)). B: SEM (scale bar 10 µm) surface image of PPy(pot, 0.75 V) with inset of cross-section image, Figure S6: Linear actuation of PPy(mirr) (■) in 0.1 M TBACF3SO3 PC electrolyte in potential ranges 1 V to −1 V showing strain against frequencies (0.00417 Hz to 0.1 Hz).
