# Supplementary material for: Antagonist Concepts of Polypyrrole Actuators: Bending Hybrid Actuator and Mirrored Trilayer Linear Actuator
Source: Polymers (Basel). 2021 Mar 11;13(6):861. doi: 10.3390/polym13060861 (PMC7999340; doi:10.3390/polym13060861)
Supplement: Supplementary file 1 [file polymers-13-00861-s001.pdf]

Supplementary

Antagonist concepts of polypyrrole actuators: bending hybrid actuator and mirrored trilayer linear actuator

Rudolf Kiefer[1,*], Ngoc Tuan Nguyen[2], Quoc Bao Le[1], Gholamreza Anbarjafari[3] and Tarmo Tamm[4]

[1]Conducting polymers in composites and applications Research Group, Faculty of Applied Sciences, Ton Duc Thang University, Ho Chi Minh City 700000, Vietnam

[2]Faculty of Applied Sciences, Ton Duc Thang University, Ho Chi Minh City 700000, Vietnam

[3]iCV Research Lab, Institute of Technology, University of Tartu, Tartu 50411, Estonia

[4]Intelligent Materials and Systems Lab, Institute of Technology, University of Tartu, Nooruse 1, 50411 Tartu, Estonia

[*]Corresponding author, E-mail address: rudolf.kiefer@tdtu.edu.vn (R. Kiefer).

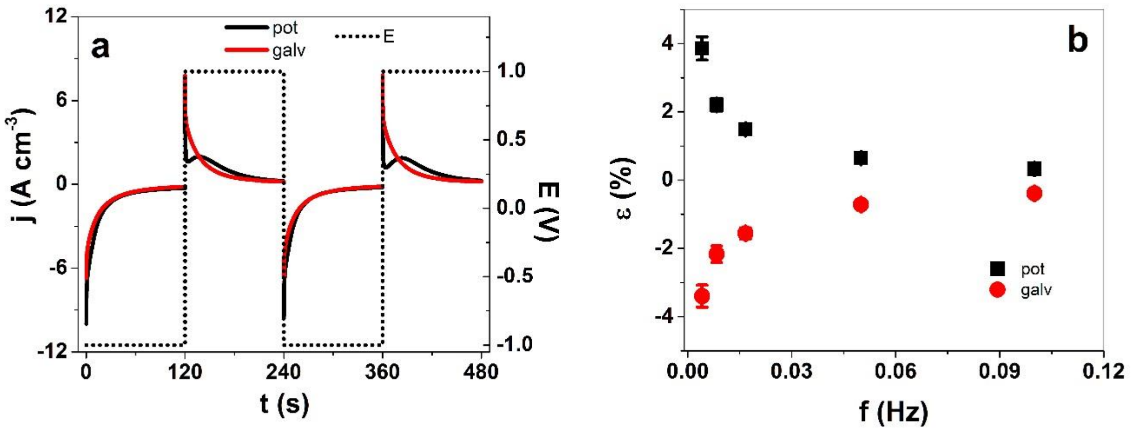

Figure S1. a: The charge density j against time t of PPy(pot) (black line) and PPy(galv) (red line) films at frequency 0.00417 Hz showing 2 subsequent cycles in $TBACF_3SO_3$ PC electrolyte against potential E (dotted) of ± 1V. The strain (positive refers to anion driven and netaive to cation-driven) against frequencies f (0.00417 Hz – 0.1Hz) of PPy(pot) (■) and PPy(galv) (●) are presented in (b).

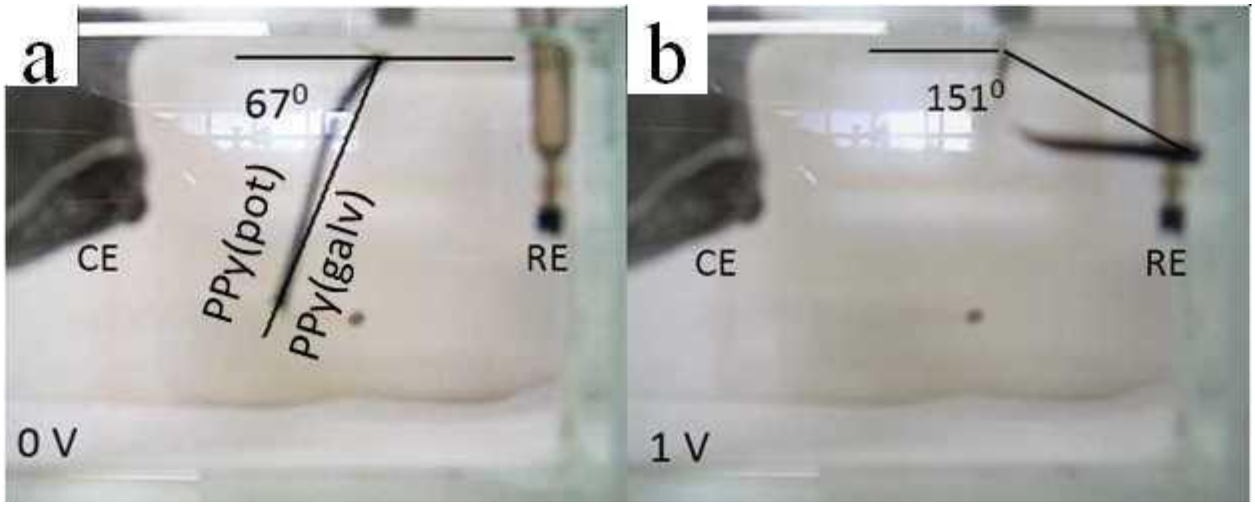

Figure 2. Images of the bending hybrid actuator (BHA) consist of PPy(pot)PPy(galv) polymerized films operated in $TBACF_3SO_3$ PC electrolyte in a three electrode cell with platinum counter electrode (CE) and a Ag/AgCl (3M KCl) reference electrode (RE) of BHA (left side the PPy(pot) and right side the PPy(galv))in potential range 0V to 1V at 0V in (a) and at +1V in (b).

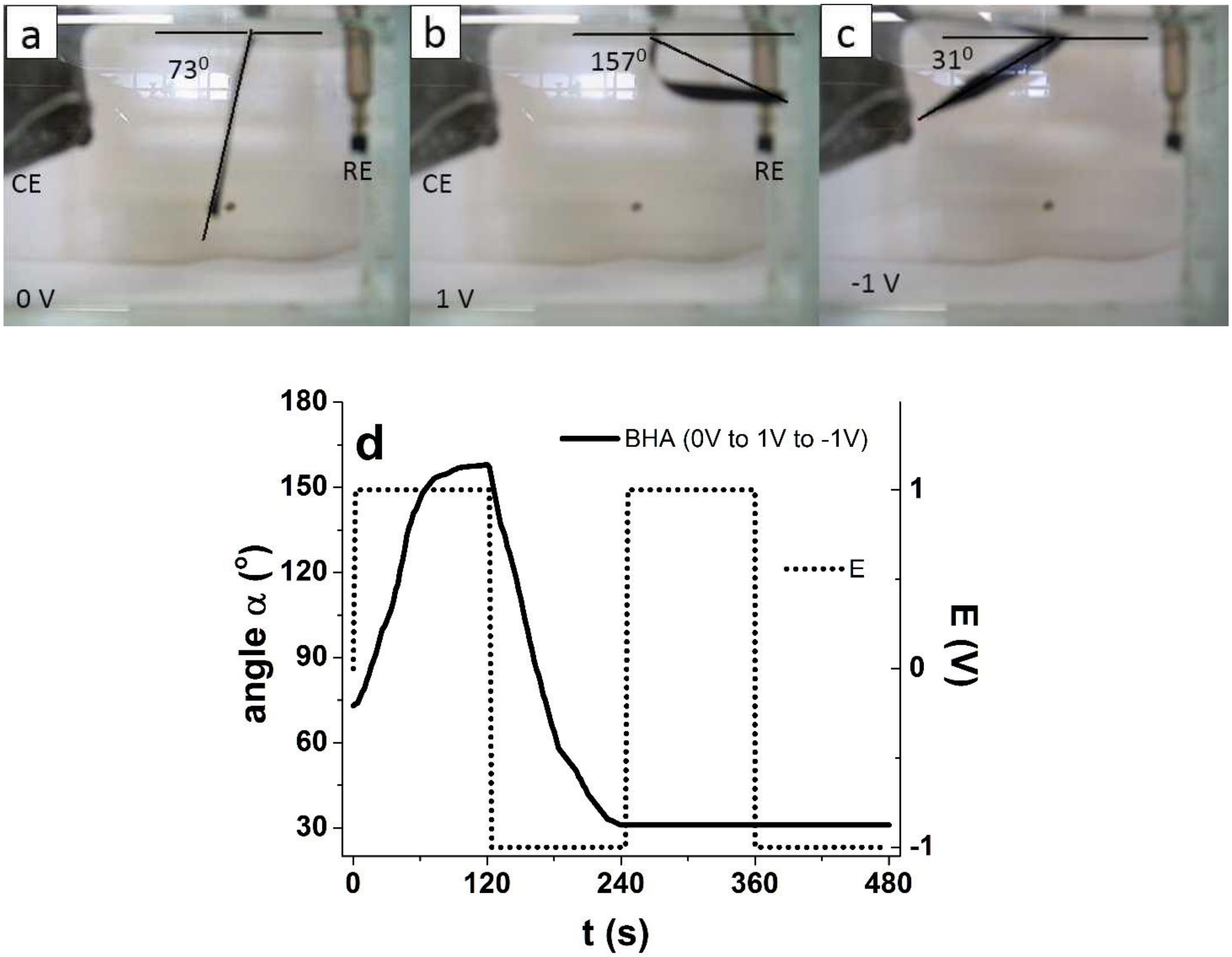

Figure S3. Images of BHA in square wave step measurements at applied frequency of 4.17mHz showing images in a: at 0V, b: at 1V and c: at -1V. The bending displacement in angle against the time t are shown in (d) with the applied potential E.

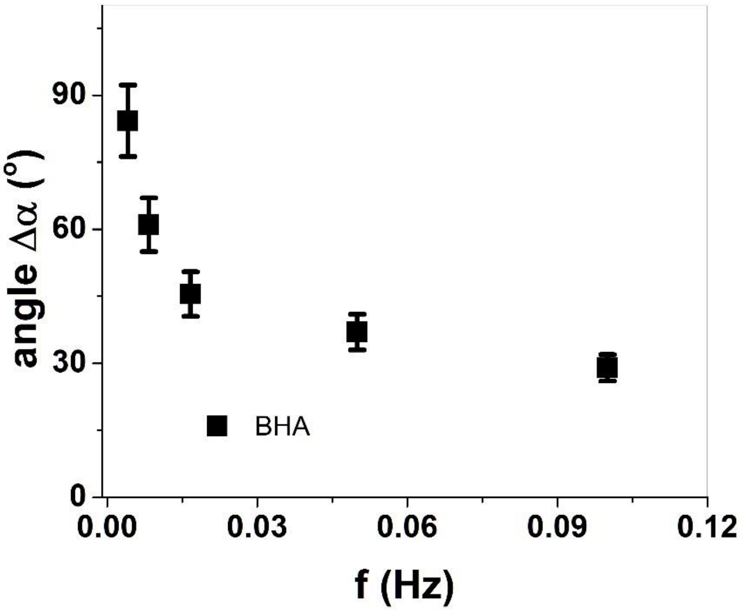

Figure S4. The displacement difference in angle $\Delta\alpha$ against frequencies f (4.17 mHz – 1 Hz) in potential range 1V to 0V are shown of the BHA samples in 0.1M $TBACF_3SO_3$ PC electrolyte.

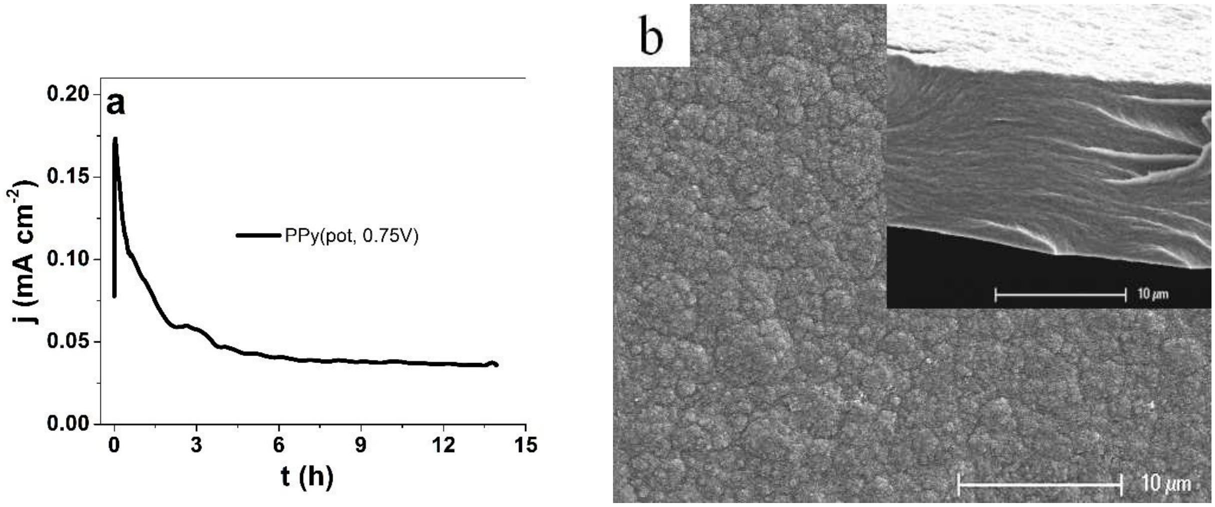

Figure S5. a: Current density time curve of PPy polymerized potentiostatically at 0.75 (at -20°C, in 0.1 M pyrrole and 0.1 M $TBACF_3SO_3$ PC) against Ag/AgCl wire (equal 0.9V against Ag/AgCl (3M KCl)). B: SEM (scale bar 10 μm) surface image of PPy(pot, 0.75V) with inset of cross-section image.

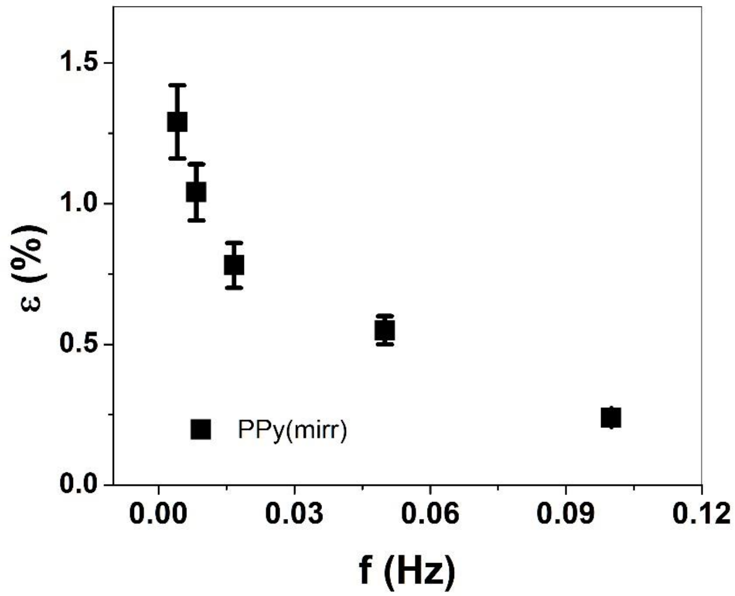

Figure S6. Linear actuation of PPy(mirr) (■) in 0.1 M $TBACF_3SO_3$ PC electrolyte in potential ranges 1V to -1V showing strain ε against frequencies (0.00417 Hz to 0.1 Hz).
